# Rapamycin Attenuates the Progression of Tau Pathology in P301S Tau Transgenic Mice

**DOI:** 10.1371/journal.pone.0062459

**Published:** 2013-05-07

**Authors:** Sefika Ozcelik, Graham Fraser, Perrine Castets, Véronique Schaeffer, Zhiva Skachokova, Karin Breu, Florence Clavaguera, Michael Sinnreich, Ludwig Kappos, Michel Goedert, Markus Tolnay, David Theo Winkler

**Affiliations:** 1 Institute of Pathology, University Hospital Basel, Basel, Switzerland; 2 Department of Neurology, University Hospital Basel, Basel, Switzerland; 3 MRC, Laboratory of Molecular Biology, Cambridge, United Kingdom; 4 Neuromuscular Research Centre, Department of Biomedicine, University Hospital Basel, Basel, Switzerland; Brigham and Women’s Hospital, Harvard Medical School, United States of America

## Abstract

Altered autophagy contributes to the pathogenesis of Alzheimer’s disease and other tauopathies, for which curative treatment options are still lacking. We have recently shown that trehalose reduces tau pathology in a tauopathy mouse model by stimulation of autophagy. Here, we studied the effect of the autophagy inducing drug rapamycin on the progression of tau pathology in P301S mutant tau transgenic mice. Rapamycin treatment resulted in a significant reduction in cortical tau tangles, less tau hyperphosphorylation, and lowered levels of insoluble tau in the forebrain. The favourable effect of rapamycin on tau pathology was paralleled by a qualitative reduction in astrogliosis. These effects were visible with early preventive or late treatment. We further noted an accumulation of the autophagy associated proteins p62 and LC3 in aged tangle bearing P301S mice that was lowered upon rapamycin treatment. Thus, rapamycin treatment defers the progression of tau pathology in a tauopathy animal model and autophagy stimulation may constitute a therapeutic approach for patients suffering from tauopathies.

## Introduction

Alzheimer’s disease (AD) and fronto-temporal dementia with tau inclusions (FTD-T) are the most frequent types of dementia [1]. They are characterized by intraneuronal accumulation, hyperphosphorylation and aggregation of tau protein. Despite of intense research efforts, causative treatments are still lacking [2] and the pathogenesis of sporadic AD and FTD-T has yet remained only partly understood. Autophagy dysfunction however is known to contribute to the evolution of different neurodegenerative proteinopathies including tauopathies [3], [4], [5], [6].

We have recently reported beneficial effects of autophagy activation by trehalose on tau pathology *in vivo* [7], and others have shown similar effects *in vitro* [8]. Autophagy can be pharmacologically stimulated by the FDA approved drug rapamycin that inhibits the mammalian target of rapamycin complex 1 (mTORC1) and thereby facilitates the formation of autophagosomes. First studies investigating the use of rapamycin for the cure of neurodegenerative disorders in transgenic mouse models reported reduced neuronal protein aggregation following rapamycin administration in a murine model of Huntington’s disease [9], a triple transgenic model of Alzheimer’s disease [10], [11], and recently in a model of spinocerebellar ataxia type 3 [12].

We here studied the effect of rapamycin on tau pathology in a pure tauopathy mouse model. We find a significant reduction of cortical tau tangle pathology in P301S mice after long- and short-term rapamycin treatment. Furthermore, astrogliosis was reduced and accumulation of the autophagy associated proteins p62 and LC3 in aged tangle bearing P301S mice was lowered. The FDA-approved drug rapamycin might thus be evaluated as a therapeutic approach for tauopathies, in particular for patients suffering from hereditary tauopathies.

## Materials and Methods

### Transgenic Mice

A total of 49 homozygous P301S tau transgenic and non-transgenic control mice ranging from 3 weeks to 5.5 months of age were included in the present study. Generation of P301S transgenic mice overexpressing the shortest human four-repeat tau isoform (0N4R) under the control of a neuron-specific Thy-1.2 promoter element has been described previously [13]. This study was carried out in strict accordance with the recommendations in the Guide for the Care and Use of Laboratory Animals of the Swiss Federal Veterinary Office. The protocol was approved by the Committee on the Ethics of Animal Experiments of the University of Basel and the Federal Veterinary Office of the Kanton Basel-Stadt (Permit Number: 2364).

### Rapamycin Treatment

In brief, P301S mice were treated twice weekly intraperitoneally with 15 mg rapamycin per kg body weight or vehicle from the age of 3 weeks to 5.5 months of age (group 5-months treatment, 5MT; n = 6 rapamycin; n = 5 vehicle), and from 3 months to 4.5 months of age (6-weeks treatment, 6WT; 6/6). For a detailed study outline including further control groups see Fig. S1.

Rapamycin powder (LC Laboratories, Woburn, MA) was dissolved at 20 mg/ml in ethanol and stored at −70°C. Before each administration, rapamycin was diluted in 5%Tween 80, 5% polyethylene glycol monolaurate (Sigma-Aldrich, Saint Louis, MO) [14], [15]. Vehicle contained comparable amounts of ethanol, Tween 80, and polyethylene glycol monolaurate as the rapamycin solution. Levels of rapamycin were measured by HPLC in blood and perfused brain tissue as published previously [16].

### Histology and Immunohistochemistry

Mice were deeply anaesthetized with sodium pentobarbital (100 mg/kg body weight) and transcardially perfused with cold phosphate-buffered saline (PBS). Brains were removed and one half of the brain was immersion fixed in 4% paraformaldehyde and embedded in paraffin. Sagittal serial sections of 20 µm thickness were cut with a microtome throughout the hemisphere [17]. Fibrillary tau tangle pathology was assessed by Gallyas silver staining. Immunohistochemistry on paraffin fixed sections was done according to previously published protocols [18] using the avidin–biotin–peroxidase complex method and VECTOR NovaRED kits (Vector Laboratories, Burlingame, CA) as chromogen. Samples of rapamycin and vehicle treated mice were processed pairwise in parallel. AT8 (ptau Ser202/Thr205, Pierce, Rockford, IL), AT100 (ptau Ser212/Thr214, Pierce, Rockford, IL), and GFAP (Clone Ab1; Thermo Scientific Freemont, CA) antibodies were used. Cortical GFAP staining was assessed qualitatively by three independent raters (S.O., K.B., D.W.). Blinded sets comprising every fifth 20 µm section of 5MT mice were rated from – to +++ and mean scores of the three raters were obtained (see also Fig. S2).

### Stereology

Quantification of tau tangles was performed on Gallyas silver stained sections using the Optical Fractionator [19]. In the 5MT group, cortex, hippocampus and brain stem were taken into account for stereological analysis, while in the 6WT group the quantification was limited to the cortex. AT8 positive cortical cells were quantified in both groups. In brief, every 5th section of 20 µm thickness throughout the left hemisphere was assessed using a Zeiss Axioplan microscope and StereoInvestigator software (Version 9.14; MicroBrightField, Williston, VT).

### Sarkosyl Extraction and Western Blot Analysis

Following PBS perfusion, one half of the mouse brain was dissected into forebrain and brain stem and immediately frozen in liquid nitrogen. Sarkosyl extraction was performed as described previously [20]. The brain tissue was homogenized in 0.5 ml of 800 mM NaCl, 10% sucrose, 10 mM tris HCl pH 7.4, 1 mM EGTA using a Kinetica polytron. Samples were centrifuged at 5000 rpm for 15 min. The supernatant was collected and sarkosyl added to 1% for 1 hour, shaking. Samples were then centrifuged at 80,000 rpm for 30 min and the pellet resuspended in 150 µl/g of tissue 10 mM Tris HCl pH 7.4. Antibodies used for Western blotting are listed in detail including the targeted epitopes in Methods S1. In brief, we used for detection of tau: BR134 [21]; RD3 from Millipore Corporation (Billerica, MA); T49 [22], [23], a kind gift of Prof. Virginia Lee, CNDR, University of Pennsylvania School of Medicine, Philadelphia, PA; AT8 and AT100 from Pierce Biotechnology (Rockford, IL). For evaluation of mTORC1 signalling and autophagy were used: anti-S6 Ribosomal Protein (#2217), anti-Phospho-S6 Ribosomal Protein Ser235/236 (#2211), anti-LC3B (#2775), from Cell Signaling Technology (Danvers, MA); anti-Phospho-S6 Ribosomal anti-p62 (GP62-C) from Progen Biotechnik (Heidelberg, Germany).

### Statistical Analysis

Statistical analysis was performed using IBM® SPSS® Statistics Version 19. Biochemical data of tau Western blots was subjected to unpaired T-tests and T-tests adjusted for unequal variances (Welch-Test), yielding both similar results. Holm-Bonferroni corrections were applied. Biochemical data of p62 and LC3-levels was analyzed by ANOVA. Stereological samples of vehicle and rapamycin treated mice were stained in parallel. Accordingly, the pairwise reduction of Gallyas or AT8 positive counts was analysed using one-sample T-tests per brain region. Significant p-values adjusted for multiple comparisons by the Holm-Bonferroni method are reported and outlined in all figures as follows: * = p<0.05, ** = p<0.01, ***p = <0.001. The mean and SD are indicated in all figures.

## Results

**Figure 1:**
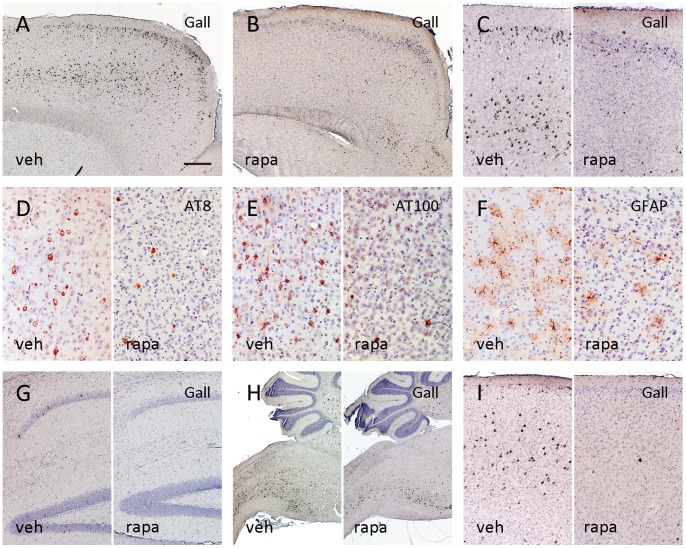
The extensive cortical tau tangle pathology present in 5.5 months old vehicle (veh) treated P301S mice (A) was widely attenuated in long-term rapamycin (rapa) treated mice (B). The lowering in tangle formation was most pronounced in the motor cortex (C: left vehicle treated/right rapamycin) and associated with reduced pathological tau hyperphosphorylation at the AT8 and AT100 epitopes (D, E). In parallel, cortical astrogliosis was diminished following rapamycin treatment (F). While there was a trend towards a reduction of the sparse tangles in the hippocampus, the advanced tau pathology in the brain stem however was not significantly ameliorated by rapamycin (G, H). Short-term treatment at 3 months of age for 6 weeks again resulted in a marked reduction of cortical tangles (I) (A–H: 5MT group; I: 6WT. A–C, G–I: Gallyas silver stain; D: AT8 IHC; E: AT100 IHC; F: GFAP IHC. Bar in A equals 300 µm in A and B, 150 µm in C, G and I, 75 µm in D–F, 600 µm in H).

Aged vehicle treated P301S tau transgenic mice developed extensive tau pathology, spreading throughout the central nervous system including the forebrain, comparable with previous reports [13] (Fig. 1A). Preventive long-term rapamycin administration initiated at 3 weeks of age resulted in a marked reduction of cortical tau tangles at age of 5.5 months (Fig. 1B). While in vehicle treated mice, abundant Gallyas stain positive tangle pathology was seen throughout the cortex, pronounced in rostral motor cortex, only few isolated tangles formed in the cortex of rapamycin treated 5MT P301S mice (Fig. 1C). In parallel, cortical tau phosphorylation at the early hyperphosphorylated AT8 epitope and the late AT100 epitope was diminished by rapamycin treatment (1D, E). With the evolution of tau tangle pathology, P301S mice develop progressive astrogliosis [24]. In parallel to the marked reduction in cortical tangle load, we noted a qualitative alleviation of cortical astrogliosis in long-term rapamycin treated mice (Fig. 1F, Fig. S2).

While in the hippocampus of vehicle treated 5MT P301S mice, only sparse tangles and limited tau hyperphosphorylation were seen, there was a markedly advanced tangle formation affecting the brain stem region. Only a mild qualitative reduction of the sparse hippocampal and the extensive brain stem tau pathology became visible following rapamycin administration (Fig. 1G, H).

We further studied whether a favourable effect on tau pathology progression could also be attained when treatment was started only after the onset of tau hyperphosphorylation in P301S mice. A short 6 weeks treatment with rapamycin was initiated at 3 months of age (6WT group) and again resulted in a notable reduction of cortical tau hyperphosphorylation and tangles (Fig. 1I).

**Figure 2:**
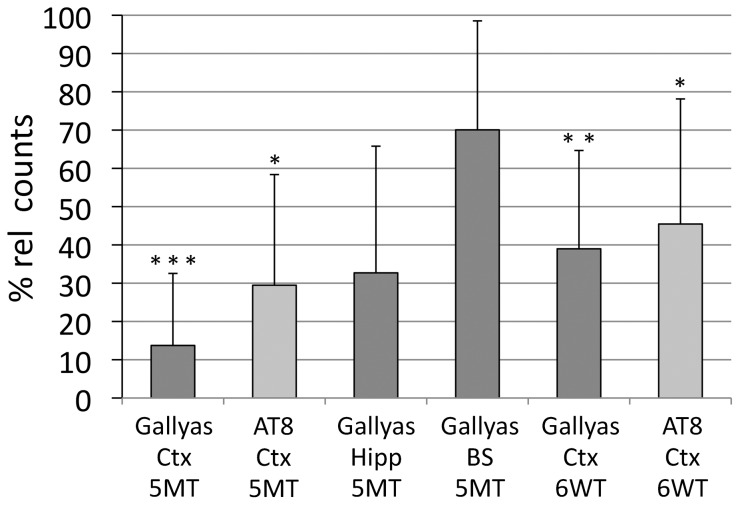
After long-term rapamycin treatment (n = 6), unbiased stereology confirmed a significant reduction of cortical tau tangles to only 14% of the amount of tangles seen in vehicle treated (n = 5) P301S mice (vehicle = 100%). The number of AT8 stained cells containing hyperphosphorylated tau was reduced to 30% in aged rapamycin treated mice compared to controls (100%) (5MT group). A significant attenuation of Gallyas-stained (to 39% of controls) and AT8-positive cells (to 46% of controls) was also achieved by late short-term rapamycin treatment (6WT, n = 6/6). The reduction of tangles in the hippocampus (to 33% of controls) and the brain stem (to 72% of controls) did not reach the level of significance adjusted for multiple testing. Pairwise reduction of Gallyas or AT8 positive counts was analysed using one-sample T-tests per brain region. Significant p-values, adjusted for the multiple comparisons of all 6 tested groups by the Holm-Bonferroni method, are outlined as follows: *p<0.05, **p<0.01, and ***p<0.001.

Quantitative stereological analysis confirmed a significant reduction in cortical tangles by 86% comparing long-term rapamycin treated mice to vehicle treated mice (5MT group, n = 5, Gallyas positive tangle count reduced to 13.7%±18.8%, p<0.001) (Fig. 2). Cortical tau hyperphosphorylation, assessed by neuronal AT8 positivity, was reduced to 29.5±28.9% (p = 0.02) in long-term rapamycin treated mice (5MT). While a trend towards a reduction of tau tangles was noted in the hippocampus (p = 0.05), the lowering of the marked tangle pathology observed in the brain stem region did not reach statistical significance (Fig. 2).

The short 6 weeks treatment started at 3 months of age significantly reduced cortical tangles (6WT group, n = 6, tangle count reduced to 38.9%±25.7%, p = 0.004) and lowered tau hyperphosphorylation at the AT8 epitope (reduction to 45.5±32.7%; p = 0.04; Fig. 2).

**Figure 3:**
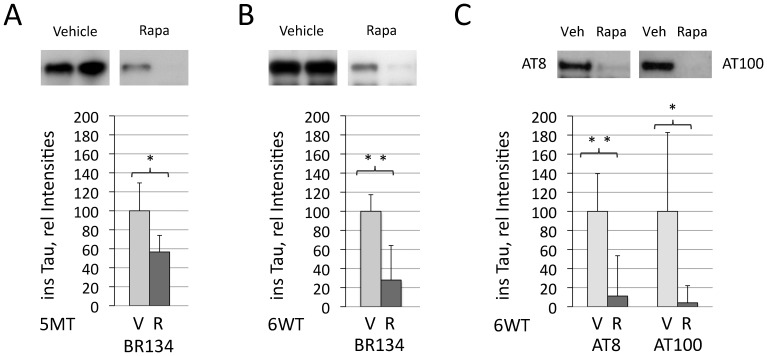
Levels of sarkosyl extracted insoluble tau were significantly reduced in the forebrain of P301S mice after 5 months of long-term rapamycin treatment (R, n = 6) when compared to vehicle treated mice (V, n = 5) (A, 5MT group; Western blot using BR134 antibody). A comparable lowering of insoluble tau was obtained by late short-term rapamycin administration over 6 weeks (B; 6WT group, n = 6/6). In parallel, the accumulation of tau hyperphosphorylated at the AT8 and AT100 epitopes was significantly lowered (C; 6WT group, n = 6/6). Quantification of tau Western blots was subjected to unpaired T-tests and T-tests adjusted for unequal variances (Welch-Test), yielding both similar results. *p<0.05 and **p<0.01.

In parallel to the observed attenuation in cortical tau tangle pathology in the stereological assessment, biochemical analysis revealed a significant reduction of sarkosyl insoluble tau in the forebrain of long- and short-term treated P301S mice (5MT: rapamycin group: percentage of vehicle treated mice: 56.7±17.5%, p = 0.03, Fig. 3A; 6WT: 28.0±36.2%, p = 0.004, Fig. 3B). Tau hyperphosphorylated at AT8 and AT100 was significantly lowered upon 6 weeks of rapamycin treatment as measured by Western blotting (6WT: rapamycin group: percentage of vehicle treated mice: AT8∶11.2±42.3%, p = 0.004; AT100∶4.1±17.9%, p = 0.04, Fig. 3C). In contrast, no decrease of forebrain soluble tau levels was noted in these aged mice, and no acute suppression of soluble tau protein generation occurred after rapamycin administration in pretangle P301S mice (Fig. S3A). Endogenous murine tau furthermore remained unchanged after acute and chronic rapamycin administration in P301S mice as assessed by the mouse tau specific antibody T49 [22] (Fig. S3B).

**Figure 4:**
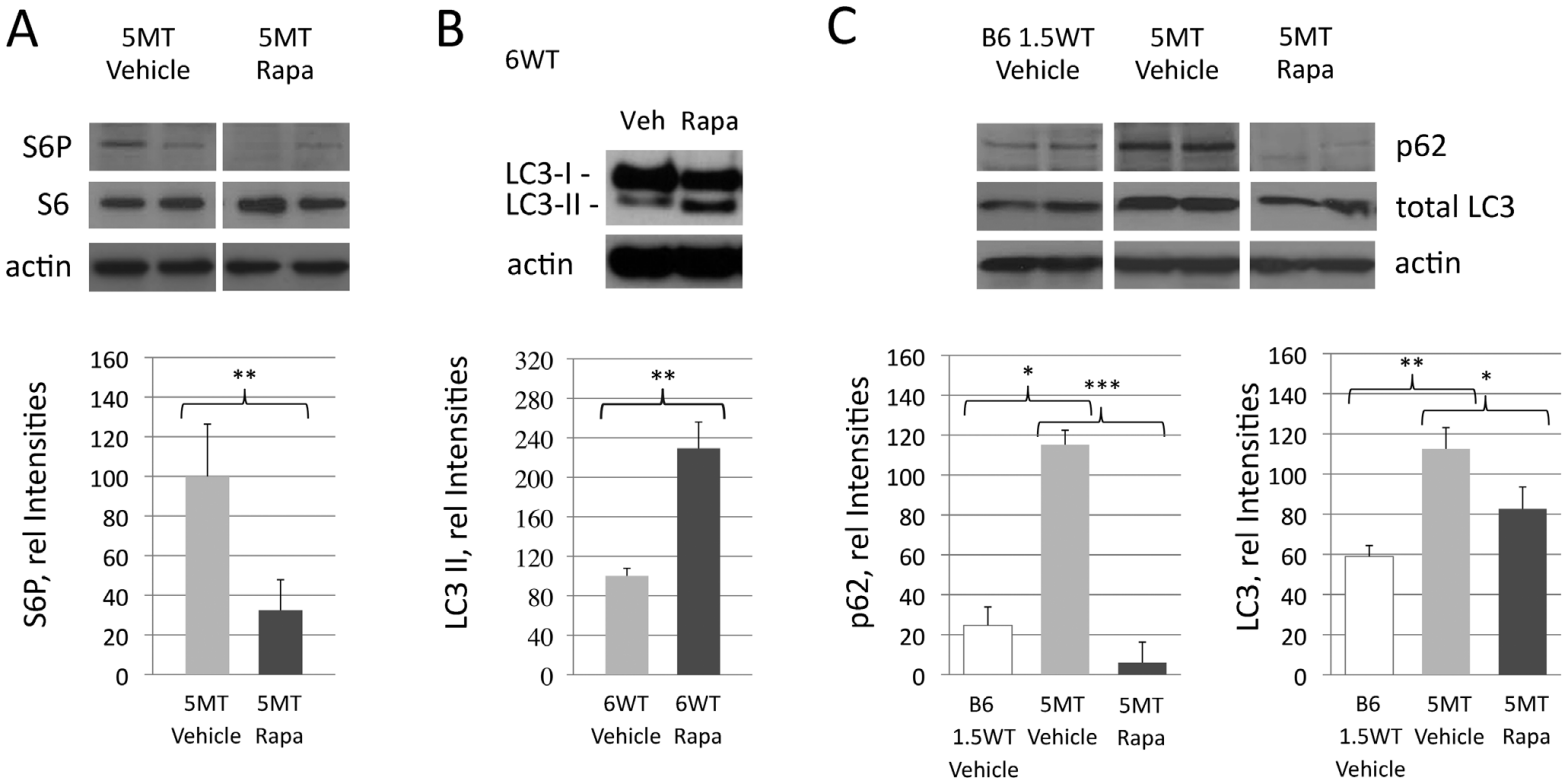
Consistent with cerebral mTOR inhibition, phosphorylation of S6 (S6P) was significantly reduced following rapamycin administration (A, 5MT group, n = 4/4). Compatible with an induced autophagy pathway, LC3II levels were increased upon rapamycin treatment (B, 6WT group, n = 6/6). High levels of the autophagy associated proteins p62 and LC3 were measured in aged vehicle treated P301S transgenic mice (C; 5MT Vehicle, n = 4). This accumulation of p62 and LC3 was prevented by long-term rapamycin administration, pointing towards a restored autophagic flux (C; 5MT Rapa, n = 4). Forebrain tissue was used and data was analyzed by ANOVA. *p<0.05, **p<0.01, and ***p<0.001.

High levels of rapamycin were measured in the brain following its intraperitoneal administration, in confirmation that rapamycin penetrates the blood-brain barrier (Fig. S4A). Rapamycin induced inhibition of the mTORC1 pathway in the brain of treated mice resulted in significantly reduced phosphorylation of ribosomal S6 protein (S6) (5MT, Fig. 4A). S6 suppression by rapamycin was comparable in the forebrain and the brain stem (Fig. S4B).

In line with the activation of autophagy by rapamycin, a significant increase in LC3II by 229% was found in rapamycin treated P301S mice (6WT, p = 0.02, Fig. 4B). High levels of the autophagy associated proteins p62 and LC3 in a set of vehicle treated old tangle bearing P301S mice furthermore pointed towards a disturbed autophagy flux in our tauopathy model. This accumulation of p62 and LC3 was prevented by rapamycin treatment (Fig 4C).

## Discussion

We report a significant alleviation of cortical tau pathology in a murine tauopathy model following long- and short-term administration of the autophagy inducing drug rapamycin. Alzheimer’s disease (AD) and other tauopathies including fronto-temporal dementia with tau pathology (FTD-T) constitute the most prevalent forms of neurodegenerative disorders [1]. Despite a broad range of concepts, only few tau targeting approaches such as tau directed immunotherapy [25], [26] and administration of lithium chloride [27], sodium selenate [28] or methylene blue [29] have been effective *in vivo.*

Disturbed autophagy is known to be involved in the pathogenesis of AD [4], [30]. We have recently shown that trehalose alleviates tau pathology by autophagy stimulation *in vivo* [7]. A beneficial effect of autophagy induction on amyloid-β and associated tau pathology has furthermore been found in a triple transgenic mouse model of AD [10], [11].

We here studied the effect of the FDA approved drug rapamycin on tau pathology in absence of amyloid-β pathology, using the P301S mutant tau transgenic mouse model. Vehicle treated P301S mice develop extensive tau pathology with tau redistribution towards the cell body and dendrites, early tau hyperphosphorylation at 4–6 weeks of age, and subsequently, at ages of 3 months, progressive aggregation of tau into tangles. We show that long-term rapamycin treatment reduces cortical tau tangle burden by more than 80% and levels of sarkosyl insoluble tau in the forebrain by 70%. In parallel to the attenuation of tau pathology, we note reduced astrogliosis following rapamycin administration. Rapamycin is also able to alleviate tau tangle pathology when a short-term treatment is started after tau hyperphosphorylation has already been initiated. These findings extend previous observations in rodent models on beneficial effects of rapamycin for cerebral proteinopathies e.g. polyglutamine disorders [9], [12] and the amyloid-β cascade [10] to tauopathies.

Comparably to trehalose treatment [7], rapamycin does not fully prevent the formation of tau pathology in P301S mice. Particularly in the brain stem, the persisting tangle formation might be caused by the extensive basal tau pathology present in this brain region in P301S mice that could overrun the benefical effect of autophagy induction in this brain area [13]. Rapamycin treatment thus seems to be subjected to a ceiling effect that does not allow coping with a very high tau load. As a limitation of our present study, the rapidly progressive brain stem pathology furthermore precludes the observation of a significant clinical improvement in the P301S model. Detailed analysis of behavioural and functional parameters including the characterization of early effects on tau metabolism following rapamycin treatment should therefore be addressed in future studies.

Prevention from protein aggregation by rapamycin in different models of neurodegenerative disorders has mainly been attributed to its autophagy inducing property [5], [10]. Here we show that intraperitoneally administrated rapamycin penetrates the blood-brain-barrier in mice. It reaches effective levels in the brain to inhibit mTOR and stimulate autophagy. Our analysis of autophagic flux is limited by the *ex vivo* nature of our specimen and the long-term treatment effects. However, in vehicle treated, tangle bearing P301S mice, we observe an accumulation of LC3 protein and the autophagy substrate protein p62, similar to reports on findings in human tauopathy patients’ brains [31]. Lowered levels of p62 and LC3 in our rapamycin treated P301S mice thus may point towards a restoration of the autophagic flux, comparable to a recent observation in APP transgenic, amyloid-ß depositing CRND8 mice [32]. Besides autophagy stimulation, rapamycin can attenuate tauopathy progression also by its immunosuppressive properties. The later mechanism may underlie the observed reduction in astrogliosis, as tau associated gliosis has earlier been reported to be responsive to immunosuppression [33]. Rapamycin has furthermore been shown to modulate tau phosphorylation *in vitro* during neuronal development [34], [35]. A favorable effect on tau phosphorylation may therefore contribute to the attenuation of tau pathology in our model. It has furthermore been reported that rapamycin can inhibit protein synthesis [36]. We however see no reduction in endogenous mouse tau nor in transgenic human tau following short-term rapamycin administration, precluding that the observed favourable effects on tau pathology evolution are primarily based on a reduced generation of tau in our model.

Rapamycin is an established FDA-approved drug. Its use as an mTOR inhibitor for the treatment of tuberous sclerosis has recently been translated from transgenic mouse models to man [37]. The beneficial effects of rapamycin on the progression of tau pathology in our murine model may encourage the development of autophagy inducing agents for patients suffering from tauopathies.

## Supporting Information

Figure S1 Scheme of the study indicating the treatment schedules of the different groups of rapamycin (R) or vehicle (V) treated P301S mutant tau transgenic mice and non-transgenic C57BL/6J mice. P301S mice were treated twice weekly intraperitoneally with 15 mg rapamycin per kg body weight or vehicle from 3 weeks to 5.5 months of age (group 5-months treatment, 5MT; n = 6 rapamycin; n = 5 vehicle), and from 3 months to 4.5 months of age (6-weeks treatment, 6WT; 6/6). Additional P301S mice were treated at age of 3 months for 1.5 weeks in order to analyze the immediate effects on soluble tau levels (1.5-weeks-treatment, 1.5WT; 5/5). Furthermore, non-transgenic C57BL/6J mice were treated accordingly at the age of 3 months for 1.5 weeks (B6-1.5WT; 2/2). A total of 8 adult C57BL/6J mice (4 rapamycin, 4 vehicle) have been used to measure the levels of rapamycin in blood and brain (B6 sir; 4/4). A total of 4 additional rapamycin treated mice died during the experiments and could therefore not be included for data collection.
(PDF)

Figure S2 For the qualitative assessment of astrogliosis in long-term rapamycin treated mice, blinded sets comprising every 5^th^ 20 µm section of 5MT mice were rated from – (A), + (B), ++ (C) to +++ (D) by three independent raters (S.O., K.B., D.W.). The median rating of the GFAP stainings of all sections was listed per brain region and mouse for a qualitative comparison of the 5 vehicle treated to the 6 rapamycin treated mice (E).
(TIF)

Figure S3 Forebrain levels of soluble tau protein remained unchanged after both, 5 months or 6 weeks of rapamycin treatment (A; 5 MT, p = 0.35; 6 WT, p = 0.18). We also analyzed the immediate effects of a short rapamycin treatment of 1.5 weeks duration on forebrain tau levels in pretangle P301S mice. There again was no reduction of soluble tau by rapamycin treatment (A; 1.5 WT, p = 0.53). Furthermore, unchanged levels of mouse tau following rapamycin administration indicate that there is no suppression of endogenous tau synthesis by rapamycin in our model (D; T49 antibody (kindly provided by Prof. V. Lee); 1.5 WT, p = 0.20; 6 WT, p = 0.86).
(TIF)

Figure S4 Intraperitoneal rapamycin administration resulted in high cerebral rapamycin levels as measured by HPLC. Similar levels were achieved in the forebrain and the brain stem (A; B6 1.5 WT; FB = forebrain, BS: brain stem, BL: blood). Consistent with cerebral mTOR inhibition, Western blotting of forebrain tissue showed significantly reduced phosphorylation of S6 following rapamycin administration (see Fig. 4A). Suppression of the phosphorylation of S6 (S6P) was comparable in brain stem and forebrain tissue, compatible with a similar effect of rapamycin on mTOR in both brain regions.
(TIF)

Methods S1 (DOCX)
