# Supplementary figures and images for: Rapamycin Attenuates the Progression of Tau Pathology in P301S Tau Transgenic Mice
Source: PLoS One. 2013 May 7;8(5):e62459. doi: 10.1371/journal.pone.0062459 (PMC3646815; doi:10.1371/journal.pone.0062459)

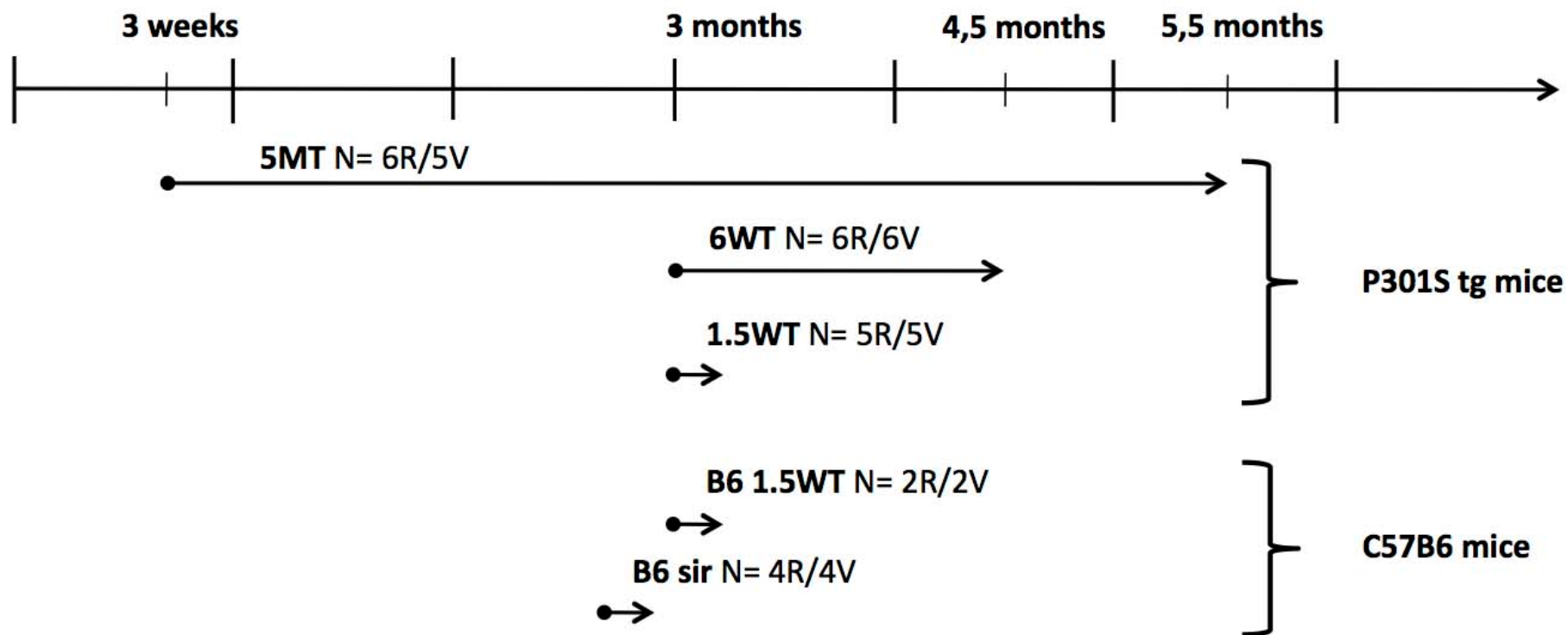

Supplement: Figure S1 — Scheme of the study indicating the treatment schedules of the different groups of rapamycin (R) or vehicle (V) treated P301S mutant tau transgenic mice and non-transgenic C57BL/6J mice. P301S mice were treated twice weekly intraperitoneally with 15 mg rapamycin per kg body weight or vehicle from 3 weeks to 5.5 months of age (group 5-months treatment, 5MT; n = 6 rapamycin; n = 5 vehicle), and from 3 months to 4.5 months of age (6-weeks treatment, 6WT; 6/6). Additional P301S mice were treated at age of 3 months for 1.5 weeks in order to analyze the immediate effects on soluble tau levels (1.5-weeks-treatment, 1.5WT; 5/5). Furthermore, non-transgenic C57BL/6J mice were treated accordingly at the age of 3 months for 1.5 weeks (B6-1.5WT; 2/2). A total of 8 adult C57BL/6J mice (4 rapamycin, 4 vehicle) have been used to measure the levels of rapamycin in blood and brain (B6 sir; 4/4). A total of 4 additional rapamycin treated mice died during the experiments and could therefore not be included for data collection. (PDF) [file pone.0062459.s001.pdf]

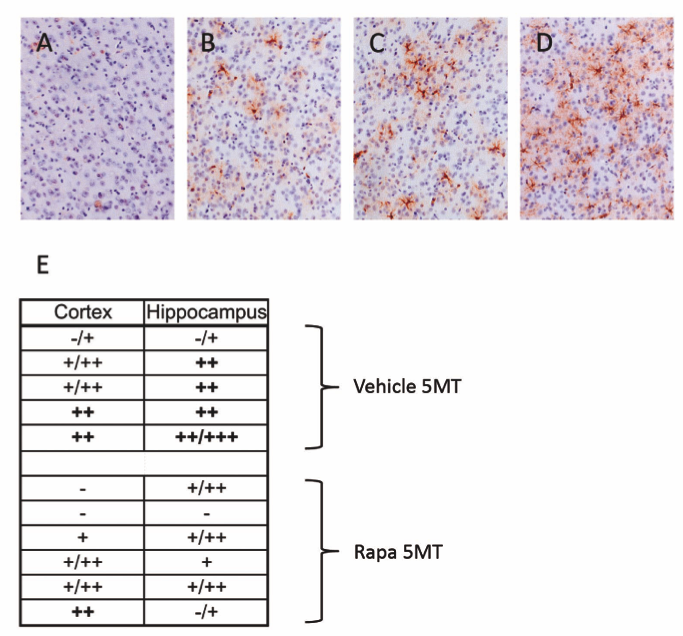

Supplement: Figure S2 — For the qualitative assessment of astrogliosis in long-term rapamycin treated mice, blinded sets comprising every 5th 20 µm section of 5MT mice were rated from – (A), + (B), ++ (C) to +++ (D) by three independent raters (S.O., K.B., D.W.). The median rating of the GFAP stainings of all sections was listed per brain region and mouse for a qualitative comparison of the 5 vehicle treated to the 6 rapamycin treated mice (E). (TIF) [file pone.0062459.s002.tif]

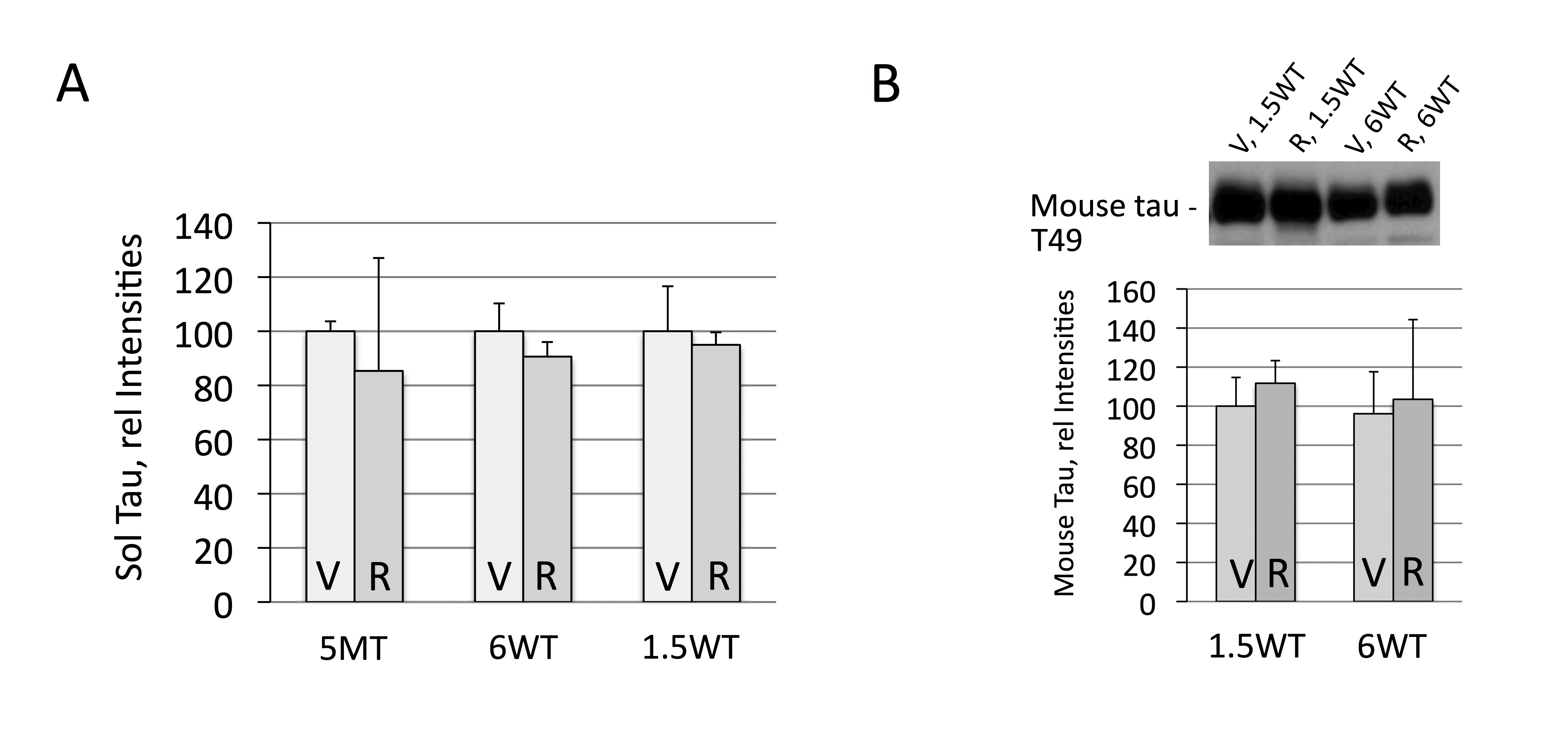

Supplement: Figure S3 — Forebrain levels of soluble tau protein remained unchanged after both, 5 months or 6 weeks of rapamycin treatment (A; 5 MT, p = 0.35; 6 WT, p = 0.18). We also analyzed the immediate effects of a short rapamycin treatment of 1.5 weeks duration on forebrain tau levels in pretangle P301S mice. There again was no reduction of soluble tau by rapamycin treatment (A; 1.5 WT, p = 0.53). Furthermore, unchanged levels of mouse tau following rapamycin administration indicate that there is no suppression of endogenous tau synthesis by rapamycin in our model (D; T49 antibody (kindly provided by Prof. V. Lee); 1.5 WT, p = 0.20; 6 WT, p = 0.86). (TIF) [file pone.0062459.s003.tif]

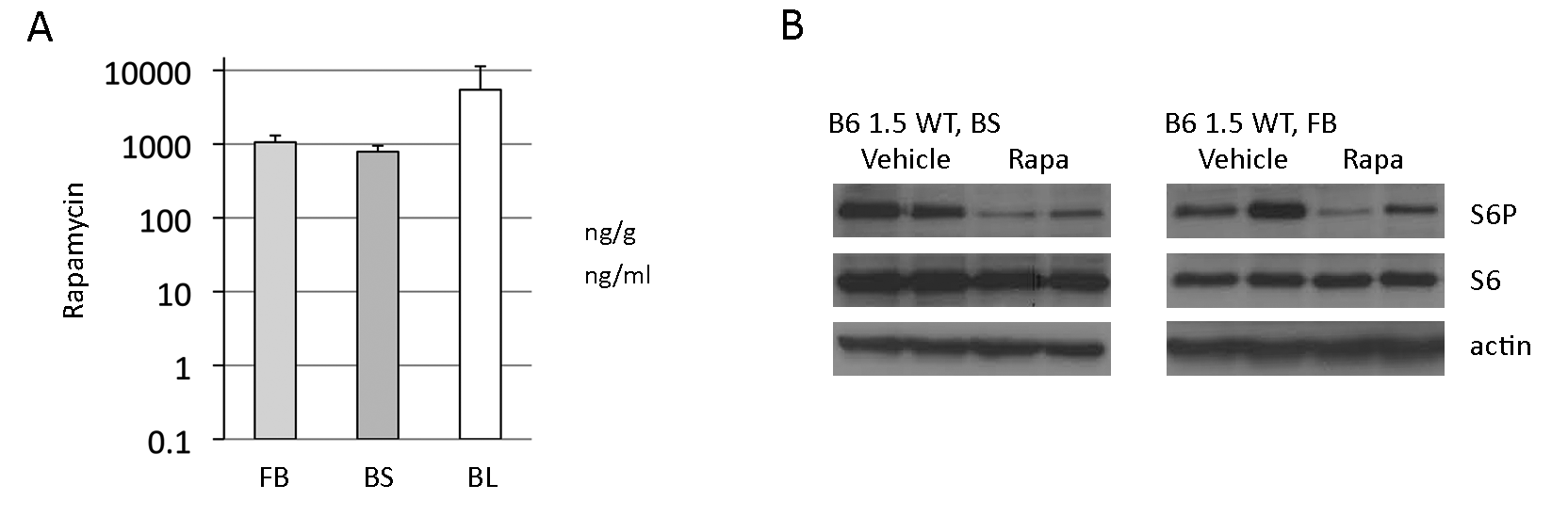

Supplement: Figure S4 — Intraperitoneal rapamycin administration resulted in high cerebral rapamycin levels as measured by HPLC. Similar levels were achieved in the forebrain and the brain stem (A; B6 1.5 WT; FB = forebrain, BS: brain stem, BL: blood). Consistent with cerebral mTOR inhibition, Western blotting of forebrain tissue showed significantly reduced phosphorylation of S6 following rapamycin administration (see Fig. 4A). Suppression of the phosphorylation of S6 (S6P) was comparable in brain stem and forebrain tissue, compatible with a similar effect of rapamycin on mTOR in both brain regions. (TIF) [file pone.0062459.s004.tif]
