# Supplementary material for: Rapamycin Attenuates the Progression of Tau Pathology in P301S Tau Transgenic Mice
Source: PLoS One. 2013 May 7;8(5):e62459. doi: 10.1371/journal.pone.0062459 (PMC3646815; doi:10.1371/journal.pone.0062459)
Supplement: Methods S1 — (DOCX) [file pone.0062459.s005.docx]

**Supplementary Methods**

*Rapamycin quantification in brain tissue and blood by HPLC*

In order to measure brain levels of rapamycin, a total of 8 C57BL/6J mice were treated for 1.5 weeks with rapamycin or vehicle (4/4). The mice then were deeply anesthetized, blood was collected in EDTA tubes following cardiac puncture, and the mice were perfused with PBS before removing and immediately freezing their brains. The forebrain halves were homogenized and subjected to HPLC measurement of rapamycin according to a previously published protocol [1]. Brain halves and blood samples of vehicle treated mice were used to calibrate the HPLC system.

*Antibodies used for Western blotting*

For Western blotting, the following antibodies were used:

1. for detection of tau: BR134 detecting the C terminus of human and murine tau independently of its phosphorylation [2], RD3 targeting aa267-316 of human 3 repeat tau and recognizing 3-repeat human and murine tau isoforms from Millipore Corporation (Billerica, MA), T49 [3,4] specific for mouse tau (kind gift of Prof. Virginia Lee, CNDR, University of Pennsylvania School of Medicine, Philadelphia, PA), AT8 against the phosphorylated Ser202 tau residue from Pierce Biotechnology (Rockford, IL), and AT100 against tau phosphorylated at Ser212 and Thr214 from Pierce Biotechnology (Rockford, IL),
2. for evaluation of mTORC1 signalling and autophagy: anti-S6 Ribosomal Protein (#2217), anti-Phospho-S6 Ribosomal Protein Ser235/236 (#2211), anti-LC3B (#2775), from Cell Signaling Technology (Danvers, MA), anti-Phospho-S6 Ribosomal anti-p62 (GP62-C) from Progen Biotechnik (Heidelberg, Germany),
3. as loading controls: anti-GAPDH ((#32233) from Santa Cruz Biotechnology (Santa Cruz, CA), and anti-beta-Actin (#A5316) from Sigma-Aldrich (Saint Louis, MO).

1. Mueller DM, Rentsch KM (2010) Sensitive quantification of sirolimus and everolimus by LC-MS/MS with online sample cleanup. J Chromatogr B Analyt Technol Biomed Life Sci 878: 1007-1012.

2. Goedert M, Spillantini MG, Jakes R, Rutherford D, Crowther RA (1989) Multiple isoforms of human microtubule-associated protein tau: sequences and localization in neurofibrillary tangles of Alzheimer's disease. Neuron 3: 519-526.

3. Mawal-Dewan M, Henley J, Van de Voorde A, Trojanowski JQ, Lee VM (1994) The phosphorylation state of tau in the developing rat brain is regulated by phosphoprotein phosphatases. J Biol Chem 269: 30981-30987.

4. Forman MS, Lal D, Zhang B, Dabir DV, Swanson E, et al. (2005) Transgenic mouse model of tau pathology in astrocytes leading to nervous system degeneration. J Neurosci 25: 3539-3550.
